# A comparative study of in vitro air–liquid interface culture models of the human airway epithelium evaluating cellular heterogeneity and gene expression at single cell resolution

**DOI:** 10.1186/s12931-023-02514-2

**Published:** 2023-08-28

**Authors:** Rachel A. Prescott, Alec P. Pankow, Maren de Vries, Keaton M. Crosse, Roosheel S. Patel, Mark Alu, Cynthia Loomis, Victor Torres, Sergei Koralov, Ellie Ivanova, Meike Dittmann, Brad R. Rosenberg

**Affiliations:** 1 Department of Microbiology, The Icahn School of Medicine at Mount Sinai, New York, NY 10029 USA; 2 Department of Microbiology, NYU Grossman School of Medicine, New York, NY 10016 USA; 3 Department of Pathology, NYU Grossman School of Medicine, New York, NY 10016 USA

**Keywords:** Airway epithelium, Innate immunity, Respiratory infection, Air–liquid interface epithelial culture, Single cell transcriptomics

## Abstract

**Background:**

The airway epithelium is composed of diverse cell types with specialized functions that mediate homeostasis and protect against respiratory pathogens. Human airway epithelial (HAE) cultures at air–liquid interface are a physiologically relevant in vitro model of this heterogeneous tissue and have enabled numerous studies of airway disease. HAE cultures are classically derived from primary epithelial cells, the relatively limited passage capacity of which can limit experimental methods and study designs. BCi-NS1.1, a previously described and widely used basal cell line engineered to express hTERT, exhibits extended passage lifespan while retaining the capacity for differentiation to HAE. However, gene expression and innate immune function in BCi-NS1.1-derived versus primary-derived HAE cultures have not been fully characterized.

**Methods:**

BCi-NS1.1-derived HAE cultures (n = 3 independent differentiations) and primary-derived HAE cultures (n = 3 distinct donors) were characterized by immunofluorescence and single cell RNA-Seq (scRNA-Seq). Innate immune functions were evaluated in response to interferon stimulation and to infection with viral and bacterial respiratory pathogens.

**Results:**

We confirm at high resolution that BCi-NS1.1- and primary-derived HAE cultures are largely similar in morphology, cell type composition, and overall gene expression patterns. While we observed cell-type specific expression differences of several interferon stimulated genes in BCi-NS1.1-derived HAE cultures, we did not observe significant differences in susceptibility to infection with influenza A virus and *Staphylococcus aureus.*

**Conclusions:**

Taken together, our results further support BCi-NS1.1-derived HAE cultures as a valuable tool for the study of airway infectious disease.

**Supplementary Information:**

The online version contains supplementary material available at 10.1186/s12931-023-02514-2.

## Background

The human airway epithelium is composed of diverse cell types which collectively function to maintain airway integrity, execute mucociliary clearance, and regulate airway immune responses. Component cell types include basal cells, the multipotent stem cells that serve as progenitors for other airway epithelial populations [1], secretory cells (encompassing both goblet cells and club cells), which secrete mucus and other anti-microbial and immunomodulatory peptides [1], and ciliated cells, which propel directional transport of mucus through coordinated ciliary activity [2]. In addition to these abundant cell types, airway basal cell precursors also give rise to rare cell types including neuroendocrine cells, tuft-like cells, and ionocytes, among others. Neuroendocrine cells sense diverse chemical and mechanical stimuli and signal these changes to the central nervous system [3, 4]. Tuft-like (or brush) cells are a chemosensory cell population that have been linked to inflammatory processes through their production of interleukin-25 and leukotrienes [5, 6]. Ionocytes, present at approximately 1–2% of airway epithelial cell frequency, express high levels of ion transporters, including CFTR, the chloride transporter dysfunctional in patients with cystic fibrosis [5, 7].

Accounting for this complexity and cellular specialization is important for physiologically relevant study of the airway epithelium in the context of infectious disease. Many respiratory viruses, such as influenza A virus (IAV) [8] and SARS-CoV-2 [9] preferentially infect ciliated cells. Human strains of IAV enter cells using α2,6-linked sialic acids (SA), present in a graded fashion along the upper airway, where they are predominantly expressed by ciliated cells and to a lesser extent, goblet (secretory) cells [10, 11]. In contrast, avian influenza strains (H5N1) utilize α2,3-linked SA which are expressed largely by alveolar type II cells in the lower respiratory tract [11]. For SARS-CoV-2, ciliated cells express both the viral receptor ACE2 and protease TMPRSS2 required for fusion at the cell membrane, and motile cilia have been shown to be required for efficient replication in in vitro airway epithelial models [2, 9]. Airway mucus, produced by secretory cells, is also critical for innate defense against respiratory infections. *Staphylococcus aureus* (*S. aureus*), *Pseudomonas aeruginosa* (*P. aeruginosa*) and *Haemophilus influenzae* (*H. influenzae*) can all elicit increased productions of mucins, which are important for airway defense but can also be detrimental in patients with underlying respiratory disease [12–14]. Experimental model systems that account for this cellular and corresponding functional diversity are important for studies of host defense to, and disease caused by, respiratory pathogens.

Primary differentiated polarized human airway epithelium (HAE) cultures at air–liquid interface are an established, physiologically relevant in vitro system used to study mechanisms of airway homeostasis and disease [15–18]. HAE cultures are classically derived from primary human bronchial epithelial cells acquired via airway biopsy, from which precursor cells can be differentiated into pseudostratified epithelia when grown on permeable supports at air–liquid interface [15]. HAE cultures recapitulate many features of the human airway epithelium in vivo, including characteristic airway cell types and secreted extracellular environment [19, 20]. As such, HAE cultures have been widely used in airway research [8, 14–18, 20–22]. An important limitation of HAE cultures derived from primary precursor cells is their restricted passage capacity before losing differentiation potential and/or becoming senescent [19]. This complicates the design of larger experiments, as sufficient material may require multiple donor sources, thereby introducing confounding genetic variation and increasing cost. Although achieved in some studies [16, 21], the limited passage capacity of primary-derived HAE cultures also poses feasibility challenges to any potential genetic manipulation, which can require extended, multi-passage selection procedures.

BCi-NS1.1 is an airway basal cell line isolated from a healthy, non-smoking male donor which has been engineered to express hTERT, thereby extending the practical lifetime of these cells up to 40 passages without losing differentiation potential [23]. In contrast, unmodified primary precursor cells can be passaged only 3–4 times before losing differentiation potential or entering senescence [19]. Previous studies have demonstrated that BCi-NS1.1 cells effectively differentiate into HAE cultures with similar morphology and cell type composition to primary HAE cultures [23]. The BCi-NS1.1 system has therefore been leveraged for a variety of applications in airway research [23–26]. However, the effects of hTERT expression on rare airway cell composition and associated gene expression programs in differentiated HAE cultures have been incompletely characterized, and any potential functional consequences for infection models with respiratory pathogens have not been directly explored. Furthermore, while BCi-NS1.1 HAE cultures should exhibit less culture-to-culture variation than HAE cultures derived from different donors, this has not been directly assessed.

Here, we perform an in-depth comparison of HAE cultures derived from BCi-NS1.1 cells (n = 3 independent differentiations) and HAE cultures derived from independent, commercially-available primary bronchial epithelial cells (n = 3 donors). Initially, we evaluate culture microarchitecture and canonical human airway epithelial cell type markers by histology and immunofluorescence labeling. We then use single cell RNA-Sequencing (scRNA-Seq) for comprehensive and unbiased assessment of constituent cell populations, differentiation states, and gene expression programs. To assess potential functional differences relevant to airway infectious disease, we evaluate HAE culture responses to infection with two common pathogens known to infect and/or colonize the airway, IAV and *S. aureus* [27, 28]. Lastly, as HAE culture systems are employed extensively in cystic fibrosis research, we evaluate the expression and function of CFTR channels in BCi-NS1.1-derived HAE cultures. Overall, we sought to thoroughly define the similarities and differences of hTERT-expressing BCi-NS1.1-derived HAE cultures and primary-derived HAE cultures to inform appropriate applications of these powerful in vitro experimental models.

## Methods

### HAE cultures

BCi-NS1.1 cells (passage 17–25) (kind gift of Dr. Ronald Crystal) and Normal Human Bronchial Epithelial cells (Lonza, cat. no. CC-2541) were seeded in Pneumacult™-Ex Plus Medium (StemCell) and passaged at least two times before plating (7 × 10^4^ cells/well) on rat-tail collagen type-I coated permeable Transwell membrane supports (Corning Inc; 6.5 mm diameter, 0.4 µm pore size) to generate HAE cultures. HAE cultures were maintained in Pneumacult™-Ex Plus Medium until confluent, then grown at air–liquid interface with Pneumacult™-ALI Medium in the basal chamber for approximately 3 weeks to form well-differentiated, polarized cultures.

### Histology, immunofluorescence labeling and imaging

Embedding, sectioning and Hematoxylin and Eosin (H&E)/Periodic acid-Schiff (PAS) Alcian blue staining of HAE cultures were performed as described previously by our laboratory[26]. For immunofluorescence labeling and imaging of HAE cultures, prepared slides were deparaffinized in Xylene (Crystalgen, cat. no. 301-038-4000) for 30 min, then rehydrated in a graded series of ethanol dilutions in water. Antigen unmasking was performed by heating slides submerged in 1X Citrate buffer (pH 6.0) (Abcam, cat. no. ab64214) in a 1.3 kW microwave for 1 min at 100% power, then for 8 min at 10% power. Slides were cooled to room temperature, washed twice with BupH Tris Buffered Saline (ThermoFisher, cat. no. 28376,) 0.05% Tween-20 (ThermoFisher, cat. no. BP337-500), and blocked with Pierce™ SuperBlock™ T20 (TBS) Blocking Buffer (ThermoFisher, cat. no. 37581) 0.5% Triton X-100 (ThermoFisher, cat. no. 85111) for 30 min. Blocking buffer was discarded and slides were incubated with primary antibody diluted in blocking buffer overnight at 4 °C. Primary antibodies included α-villin-1 diluted 1:500 (Novus Biologicals, cat. no. NBP1-85335-25ul), α-cytokeratin 5 diluted 1:500 (Abcam, cat. no. Ab75869), α-CC10 diluted 1:400 (Santa Cruz Biotechnology, cat. No. 365992), α-MUC5B diluted 1:2000 (ThermoFisher, cat. no. PA5-82342), α-MUC5AC diluted 1:1000 (ThermoFisher, cat. no MA5-12178), α-p63 diluted 1:1250 (Abcam, cat. no. Ab124762), α-FoxJ1 diluted 1:300 (ThermoFisher, cat no. 14-9965-82), and α-CFTR diluted 1:40 (ThermoFisher, cat. No. 1080-MSM9-P1). Slides were washed three times with wash buffer, then incubated with fluorescent secondary antibodies. Secondary antibodies (all diluted 1:500 in blocking buffer) were donkey anti-rabbit 647 (ThermoFisher, cat. no. A31573) or donkey anti-mouse 647 (ThermoFisher, cat. no. A31571). Slides were incubated in secondary for one hour at room temperature. Slides were washed twice with wash buffer and three times with water, then mounted with coverslips using mounting medium from Pierce™ Peroxidase IHC Detection Kit (ThermoFisher, cat. no. 36000). Slides were imaged using a Keyence BZ-X810 Fluorescent Microscope (Keyence, cat. no. BZ-X810) and images were analyzed using BZ-X800 Analyzer.

### Cell type quantification by immunofluorescence labeling

Immunofluorescence images of the villin-1, KRT5, CC10, MUC5AC and MUC5B-labeled HAE cultures were quantified using Imaris microscopy image analysis software (v9.8.2). The stitched image depicting the entire length of each HAE culture transection for each cell type stain was used for image quantification. Two technical replicate images of each biological replicate BCi-NS1.1 or primary HAE culture were used for image quantification. For each marker, signal area was determined using the surfaces creation tool and setting the intensity threshold to exclude any background as determined by HAE culture controls labeled without primary antibody. Total area of phalloidin signal was used as the total area of each HAE culture transection, and the percentage occupancy of cell type marker is represented as a percentage of this phalloidin labeled area.

### Isolation and processing of single cell suspensions from HAE cultures for scRNA-Seq

HAE cultures maintained for 3 weeks post-airlift were processed for scRNA-Seq. Single cell suspensions of HAE cultures were prepared as described previously [29]. To enable multiplexing and doublet detection, cells were labeled with “cell hashing” antibodies as described previously [30]. Briefly, approximately 200,000 cells per sample were resuspended in staining buffer (PBS, 2% BSA, and 0.01% Tween) and incubated for 10 min with Fc block (TruStain FcX, BioLegend) and FcR blocking reagent (Miltenyi Biotec). Cells were then incubated with oligonucleotide-conjugated hashing antibodies (generated in-house by the New York Genome Center as described [31]) for 30 min at 4 °C. After labelling, cells were washed three times in staining buffer. After the final wash, cells were resuspended in PBS supplemented with 0.04% BSA, filtered, and counted. Cells were pooled (6 samples per pool in equal proportions), super-loaded to the 10X Genomics Chromium Controller (approximately 25,000 cells loaded to 1 Chromium lane) and processed with the Chromium Next GEM single-cell 5′ library and gel bead kit according to manufacturer’s protocols. Hashtag-oligonucleotide (HTO) additive oligonucleotide primer was spiked into the cDNA amplification PCR, and the HTO library was prepared as described previously [31]. Gene expression and HTO libraries were pooled and sequenced in multiplex on the Illumina Novaseq 6000 system with the following read configuration: read1 28 cycles, Index read1 8 cycles, read2 91 cycles. Gene expression libraries were sequenced to an estimated depth of 32,000 read pairs per cell, and HTO libraries were sequenced to an estimated depth of 2,400 read pairs per cell.

### scRNA-Seq analysis

#### Read mapping and quantification

CellRanger count (v6.1.2, 10X Genomics Inc.) was used to assign cell barcodes and map reads to transcriptome reference (GRCh38-2020-A, GENCODE v32/Ensembl 98) with default parameters. HTO quantification was also performed using CellRanger count via feature barcode detection.

#### Data preprocessing and integration

scRNA-Seq analyses were performed in R (v4.0.3) using *Seurat* (v4.0) [32]. Samples were demultiplexed by HTO counts using the *HTODemux* function with default parameters. Demultiplexing quality control (QC) was assessed by inspection of a dimensionality reduction plot of HTO counts by t-SNE. Cell barcode × feature (gene) count matrices were filtered to exclude cells with > 18.7% mitochondrial gene content (dead or dying cells) or those with feature (gene) counts outside of the interval from 1505 to 5857 or unique molecular identifier (UMI) counts outside of the interval from 4569 to 24992, which correspond to 1.2 and 1.5 standard deviations from the mean number of counts (log), respectively. Intra-hash heterotypic doublet detection and removal were performed with *scDoubletFinder* (v1.4.0) [33]. Gene expression data were normalized with *scTransform* (v.0.3.3) [34] using top 3,000 variable genes and including a regression factor for “cell cycle difference,” determined by subtracting the G2/M score from the S score for each cell calculated using the gene sets described by Tirosh and colleagues [35]. To minimize the effect of donor-specific variation on cluster assignment, data integration was performed across all replicates using 2,000 gene features and Seurat commands *PrepSCTIntegration*, *FindIntegrationAnchors* and *IntegrateData*.

#### Data clustering and annotation

Principal component analysis (PCA) was performed on normalized, integrated data, and the first 30 principal components (PCs) were used for clustering and nearest neighbor detection. An iterative approach was used to remove low quality clusters of cells: initial clustering was performed using the graph-based Louvain algorithm with multilevel refinement [36] and a resolution parameter of 1.5. Two clusters (333 cells total) were excluded from downstream analysis due to low UMI counts and high mitochondrial content, respectively. Data were then re-clustered and assessed for stability at a range of resolutions using *Clustree* (v.0.5.0) [37]. A final resolution parameter of 1.4 was selected from these results. Clusters were annotated to canonical airway cell populations based on expression of established marker genes [4, 5, 7, 20]. Cell annotations were verified by comparing assignments to predictions obtained by label transfer from a recently published scRNA-seq dataset of similar HAE cultures [22].

### Pseudobulk differential gene expression analyses

Differential gene expression analyses were conducted on “pseudobulk” gene expression profiles using *edgeR* (v3.32.1) [38] and *scran* (v1.18.7) [39], a strategy found to provide better type I error control than individual cell-based differential expression workflows [40]. Briefly, per cell gene counts were summed to aggregate “pseudobulk” profiles for each cell population replicate, with a minimum threshold for inclusion of 25 cells. Gene expression linear models were constructed for each cell population with at least two samples per condition (BCi-NS1.1, primary) passing inclusion thresholds. Genes expressed in fewer than 10% of cells in any pseudobulk sample were excluded from differential expression tests. Differential gene expression was defined as an adjusted p*-*value < 0.05 (Benjamini-Hochberg) and an estimated log_2_ fold-change > 1.

### Pseudobulk gene set enrichment analyses

Gene set enrichment analyses were performed using *CAMERA* [41] for the molecular signatures database (MSigDB) [42] C2 canonical pathways gene set (n = 3050 sets) and HALLMARK gene set (n = 50 sets) collections. Significant set enrichment was defined as an adjusted p-value < 0.05.

### Pseudotime trajectory inference

Cell differentiation trajectories were inferred using *Slingshot *[43]. Analyses included only cells annotated along the basal-secretory axis. Principal curves were fit in integrated UMAP space and rooted to the basal cell cluster. Differential progression testing was assessed using a Kolmogorov–Smirnov Goodness of Fit Test between conditions using the *progressionTest* function with default parameters.

### Differential abundance testing

Cell abundance was modeled using *edgeR* as described [44], without normalization for total cell count. Significant differential abundance was defined as any change in cell population frequency at a p-value < 0.05.

### Infection of HAE cultures with influenza A virus

HAE cultures were washed apically twice with room temperature PBS, after which 5 × 10^6^ plaque-forming units (PFU) of influenza A/California/07/2009 virus (BEI resources, NR-13663) was added apically in 50 µl of PBS and incubated for one hour at 37 °C. HAE cultures were then washed apically once with room-temperature PBS and incubated at air–liquid interface at 37 °C for 24 h. Mock-infected cultures were treated with the same protocol as IAV-infected cultures but using only PBS. After 24 h, cultures were harvested for quantification of viral PFU, RT-qPCR, LDH quantification, imaging, or western blot analyses. For viral PFU, a 200 µl apical wash was collected from the cultures, frozen at − 80 °C and later quantified by plaque assay on MDCK cells. For RT-qPCR, cultures were washed once, and the Transwell membrane was excised and submerged in buffer RLT (Qiagen) and frozen at − 80 °C. RNA extraction was performed using the RNeasy Kit (Qiagen, cat no. 74104), cDNA synthesis using SuperScript™ III First-Strand Synthesis System (ThermoFisher, cat no. 18080051) and RT-qPCR using PowerUp™ SYBR™ Green Master Mix (ThermoFisher, cat no. A25741) with RPS11 as a housekeeping gene. For LDH quantification a 200 µl apical wash was collected from the cultures and LDH release was measured in 50 µl of apical wash in triplicate using CyQUANT™ LDH Cytotoxicity Assay (ThermoFisher, cat. no. C20300). For western blot analysis, cultures were washed once and the cells were lysed by adding 200 µl of 1 × Invitrogen™ 4X Bolt™ LDS Sample Buffer (cat. no. B0007, Fisher Scientific). Lysate was frozen at -20 °C for western blot analysis. For imaging, cultures were submerged in 4% PFA, incubated overnight at 4 °C, then transferred to PBS. Staining and imaging of infected cultures was performed as described previously by our laboratory [29].

### Infection of HAE cultures with S. aureus

*S. aureus* USA300 strain AH-LAC [45] was routinely grown at 37 °C on tryptic soy agar (TSA). For infection of HAE cultures, overnight bacterial cultures were grown in 5 mL tryptic soy broth (TSB) in 15-mL conical tubes under shaking conditions at 180 rpm and 37 °C with a 45° angle. A 1:100 dilution of overnight culture was subcultured into TSB and incubated for another 3 h as above. Bacteria were washed once with 5 mL PBS then diluted using Optical Density 600 (OD600) measurements to an OD of 0.8, corresponding to a concentration of 5 × 10^8^ CFU/mL in PBS. HAE cultures were washed apically twice with room temperature PBS, then 1 × 10^6^ CFU of AH-LAC was added apically in 50 µl of PBS and incubated for one hour at 37 °C. PBS was aspirated after 1 h of infection and HAE cultures were washed apically three times with room-temperature PBS, then incubated at 37 °C for 18 h. After 18 h, cultures were harvested for either quantification of bacterial CFU, LDH quantification or imaging. For quantification of bacterial CFU, the Transwell membrane was excised and submerged in 1% Saponin to lyse cells, and lysate was serially diluted and plated on TSA. Samples were harvested and prepared for LDH quantification and imaging as described above.

### Treatment of HAE cultures with IFN-β

For treatment with IFN-β, basolateral media was removed from the cultures and media containing 250 U (500 U/mL) human IFN-β (Millipore Sigma, cat. no. IF014, lot 3481402) was added to the basolateral chamber. IFN-β-treated cultures were incubated for 24 h at 37 °C. After 24 h, IFN-β-treated cultures were harvested and prepared for either RT-qPCR or western blot analysis as described above.

### CFTR functional assays

To assess the function of CFTR channels in hTERT-derived HAE cultures, transepithelial electrical resistance (TEER) of fully differentiated cultures was measured at baseline. Cultures were then treated with either untreated Pneumacult™ ALI basal media as a control, 0.2% DMSO as a vehicle control, or 20µM forskolin and 50µM 3-Isobutyl-1-methylxanthine (IBMX) to activate CFTR channels. TEER of cultures treated with each condition were subsequently measured at 10 min, 30 min, and 60 min post-treatment.

## Results

**Fig. 1:**
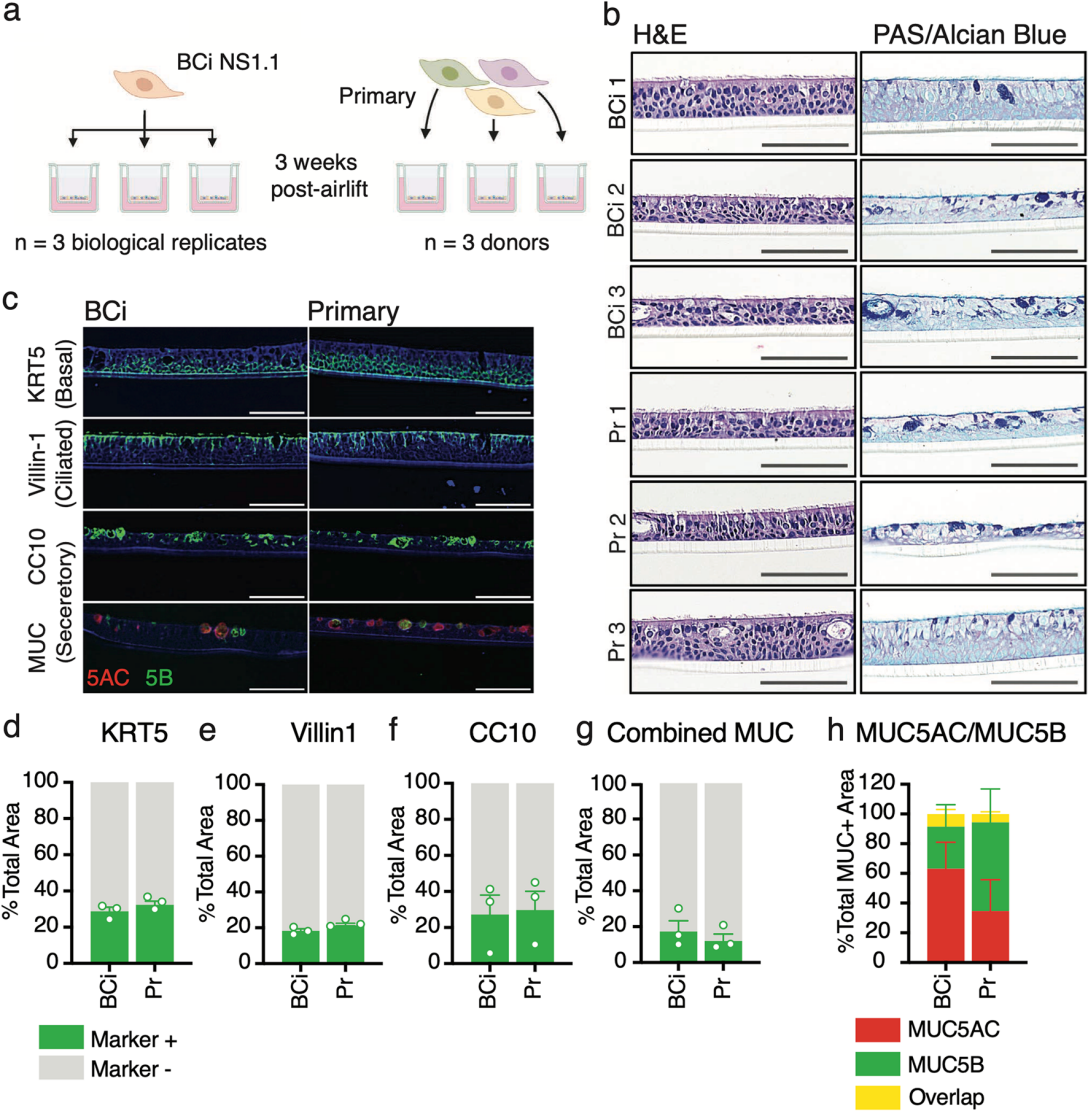
Analysis of epithelial morphology and cell types in differentiated BCi-NS1.1- and primary-derived HAE cultures. **a** Experimental design. HAE cultures were seeded from either primary cells or the hTERT-expressing airway basal cell line BCi-NS1.1. Cells were differentiated for three weeks at air–liquid interface to generate three replicates of BCi-NS1.1-HAE cultures (derived from one donor), and three HAE cultures from three separate primary donors. **b** Hematoxylin & Eosin (H&E) and Periodic Acid-Schiff (PAS)-Alcian blue staining of formalin-fixed paraffin-embedded transections of HAE cultures. All cross-sectional images are oriented with the basolateral surface of the culture at the bottom of the image and the apical surface at the top of the image. Scale bars = 150 µm **c.** Cell type marker immunofluorescence of transections of BCi-NS1.1- or primary-derived HAE cultures. DAPI (nuclei) stained in blue. Cytokeratin 5 (KRT5, basal cells), villin-1 (ciliated cells), CC10 (encoded by *SCGB1A1*, secretory cells) and MUC5B (secretory cells) labeled in green. MUC5AC (secretory cells) labeled in red. Scale bars = 150 µm. **d**–**g** Quantification for each cell type marker, KRT5 (**c**), villin-1 (**d**), CC10 (**e**), and mucins (**f**) (sum of MUC5B and MUC5AC), represented as a percentage of the total phalloidin (actin)-labeled area of the HAE cultures. **h** Quantification of cells using Imaris software labeled positive for MUC5B, MUC5AC, or both, represented as a percentage of total MUC5B and/or MUC5AC positive labeling

To assess similarities and differences between HAE cultures derived from the hTERT-expressing bronchial basal epithelial cell line BCi-NS1.1 and unmodified, primary-derived precursor cells, we generated HAE cultures from three donors of primary normal human bronchial epithelial cells, as well as three biological replicates of HAE cultures from BCi-NS1.1 (Fig. 1a).

### Both primary and BCi-NS1.1 precursor cells differentiate into pseudostratified epithelia that include basal, secretory, and ciliated cells

We first characterized the histological morphology of HAE cultures derived from BCi-NS1.1 cells and primary cells. H&E staining of HAE culture sections demonstrated that cultures derived from both BCi-NS1.1 and primary precursors adopt a polarized pseudostratified epithelial morphology with ciliated cells facing the apical side (Fig. 1b). PAS-Alcian blue staining revealed the presence of intracellular mucins (dark blue), consistent with the presence of secretory cells (Fig. 1b). Overall, individual cultures exhibited subtle differences in epithelium thickness, secretory cell frequency, and secretory cell hyperplasia, but these differences were not associated with precursor cell source.

Next, we determined the presence and intra-epithelial organization of ciliated, basal, and secretory cells by immunofluorescence microscopy. Cultures were immunolabeled for canonical cell type-specific markers: villin-1 for ciliated cells[46], cytokeratin-5 (KRT5) for basal cells[47], and CC10 (encoded by the *SCGB1A1* gene), MUC5B and MUC5AC for secretory cells [19, 20, 23, 47]. We observed KRT5 expression in small, brick-shaped cells lining the basolateral surface consistent with basal cells (Fig. 1c, Additional file 4: Fig. S1a). Image quantification of this marker identified KRT5-positive cells to account for approximately 31% of total cell area in both BCi-NS1.1- and primary-derived HAE cultures (Fig. 1d). We observed villin-1 in columnar cells lining the apical surface, consistent with ciliated cells (Fig. 1c, Additional file 4: Fig. S1b). These cells accounted for approximately 20% of total cell area in both BCi-NS1.1- and primary-derived HAE cultures (Fig. 1e). Lastly, secretory cell makers were present in morphologically heterogeneous cells, including large globular cells facing the apical surface (Fig. 1c, Additional file 4: Fig. S1c). CC10-positive cells made up on average 28% of total cell area in both BCi-NS1.1- and primary-derived HAE cultures, although the number of cells positive for this marker was more variable between cultures than either KRT5 or villin-1 (Fig. 1f). Cells positive for either MUC5AC or MUC5B generally made up less than 20% of the total cell area (Fig. 1c, g, Additional file 4: Fig. S1d). While technical constraints prohibited multiplex staining for CC10 and mucins, dual staining for MUC5B and MUC5AC revealed that most but not all MUC-positive cells expressed one of these two mucins but not both (Fig. 1c, g, Additional file 4: Fig. S1d). Although variable across cultures, the proportions of MUC5B vs MUC5AC positive cells (Fig. 1g, Additional file 4: Fig. S1d) were not associated with precursor cell source. Taken together, immunofluorescence labeling of cell-type-specific markers revealed that ciliated, basal, and secretory cells are present in both BCi-NS1.1 and primary-derived cultures at similar frequencies.

### scRNA-Seq resolves constituent cell populations of BCi-NS1.1- and primary-derived HAE cultures

**Fig. 2:**
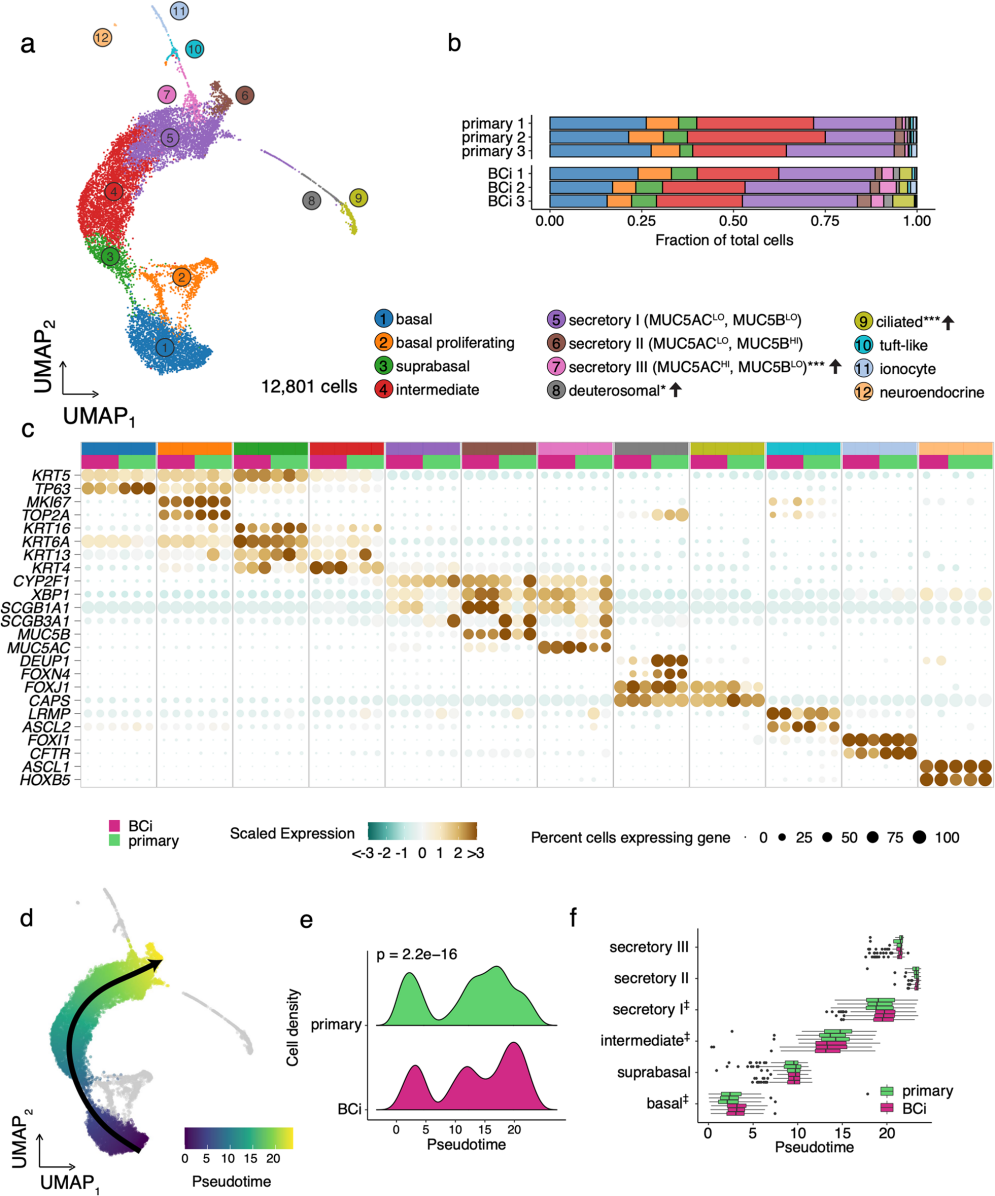
Cell annotation and pseudotime analyses of scRNA-Seq data from BCi-NS1.1 and primary–derived HAE cultures. **a** UMAP (gene expression data for n = 12,801 single cells), colored by cell population assignment. **b** Cell frequencies by biological replicate. Cell populations with significantly different frequencies (BCi-NS1.1 *versus* primary HAE cultures) are noted in population label key (*P < 0.05, **P < 0.01, ***P < 0.001)*.* **c** A dot plot summarizing the expression level (z-scaled, dot color) and percentage of cells expressing the indicated gene (dot size) of signature marker genes for each cell population per biological replicate. **d** Pseudotime trajectory lineage spanning basal-to-secretory cells (both HAE precursor groups, integrated). Proliferating cells and low frequency cell types (grey) were excluded from trajectory analysis. **e** Cell distributions for each precursor cell group along the basal-to-secretory trajectory (Kolmogorov–Smirnov Goodness of Fit Test, p = 2.2e−16). **f** Cell pseudotime values by population and scRNA-Seq replicate. Statisical testing for differential median pseudotimes for basal, intermediate and secretory I cells all reached the minimal p value of 0.1 via a Wilcoxon Rank Sum Test with 3 replicates per group (‡)

To assess the cellular composition at high resolution along with associated gene expression patterns of BCi-NS1.1- and primary-derived HAE cultures, we performed scRNA-Seq. In total, we analyzed 12,801 single cells (post quality-control filtering) from three samples of each HAE culture source (n = 3 independent differentiations of BCi-NS1.1, n = 3 different donors of primary cells). Unsupervised graph-based clustering identified 12 airway epithelial cell populations, which were annotated based on RNA expression of canonical marker genes (Fig. 2a) and label transfer predictions from a recently reported scRNA-Seq analysis of HAE cultures[22]. scRNA-Seq data included basal cells (*KRT5*^+^, *TP63*^hi^), proliferating cells (*MKI67*^+^, *TOP2A*^+^), suprabasal cells (*KRT6A*^+^, *KRT16*^+^),”intermediate “ (i.e. along the basal-to-secretory differentiation axis) cells, three populations of secretory cells (I: *MUC5AC*^lo^ *MUC5B*^lo^, II: *MUC5AC*^lo^ *MUC5B*^hi^, and III: *MUC5AC*^hi^ *MUC5B*^lo^), deuterosomal cells (*DEUP1*^+^, *FOXN4*^+^)[20], ciliated cells (*FOXJ1*^+^,

*CAPS*^+^), tuft-like cells (*LRMP*^+^, *ASCL2*^+^)[4, 7], ionocytes (*FOXI1*^+^, *CFTR*^+^)[5, 7], and neuroendocrine cells (*ASCL1*^+^, *HOXB5*^+^)(Fig. 2b). Cell representation and annotation was consistent across samples (Additional file 4: Fig. S2a). The relative frequencies of most cell populations were similar between primary and BCi-NS1.1-derived HAE cultures, with the exception of MUC5AC^hi^ secretory III cells, deuterosomal cells, and ciliated cells, which were more highly represented in BCi-NS1.1-derived HAE cultures (Fig. 2c; Additional file 1). Notably, both culture sources exhibited rare ionocytes, tuft-like cells, and neuroendocrine cells at similar frequency. Unexpectedly, we detected far fewer ciliated cells in scRNA-Seq data than by immunofluorescence (~ 2% of cells vs ~ 20% of total cell area). Based on our prior experience analyzing HAE by scRNA-Seq, we attribute this discrepancy to technical issues during sample processing resulting in the selective loss of ciliated cells prior to 10X Chromium loading. With severely reduced ciliated cell numbers, we focused the remainder of our scRNA-Seq analyses on robustly sampled basal and secretory cells. Despite these limitations, scRNA-Seq identified the expected constituent cell populations of the airway epithelium with few differences in frequency across culture types.

### Pseudotime analysis of scRNA-Seq data infers a common basal-to-secretory cell differentiation trajectory but differences in distribution of BCi-NS1.1-derived HAE cultures

As we observed subtle differences in the frequency of cell populations between primary- and BCi-NS1.1-derived HAE cultures, we investigated the possibility that these differences in cellular composition could be related to the airway cell differentiation process. To this end, we inferred differentiation trajectories by pseudotime analysis. We restricted our analysis to basal, suprabasal, intermediate, and secretory populations based on the established basal-to-secretory differentiation path [20] and to eliminate potential confounding factors from actively proliferating cells and rare cell types for which we lacked robust sampling. A single lineage was identified for both culture types: basal-to-suprabasal-to-intermediate-to-secretory (with component secretory populations ordered I, III, II) (Fig. 2d). Differential distribution testing indicated significant differences in progression along this trajectory between the culture conditions, with primary-derived HAE cultures exhibiting relatively more cells in basal and “early” secretory states in comparison to the BCi-NS1.1-derived cultures (Fig. 2e). A closer inspection of the pseudotime distribution for each cell population illustrated several shifts in the median pseudotime values: in comparison to primary-derived HAE cultures, the median basal and secretory I pseudotime values were higher in BCi-NS1.1-derived HAE cultures, while the median pseudotime value for intermediate cells was lower (Fig. 2f). This suggests that, while general cell population abundances are consistent across culture types, there exist modest but significant differences in the distribution of BCi-NS1.1-derived HAE cultures along the basal-to-secretory differentiation axis.

### HAE culture cell populations exhibit generally similar gene expression patterns across BCi-NS1.1- and primary-derived cultures

**Fig. 3:**
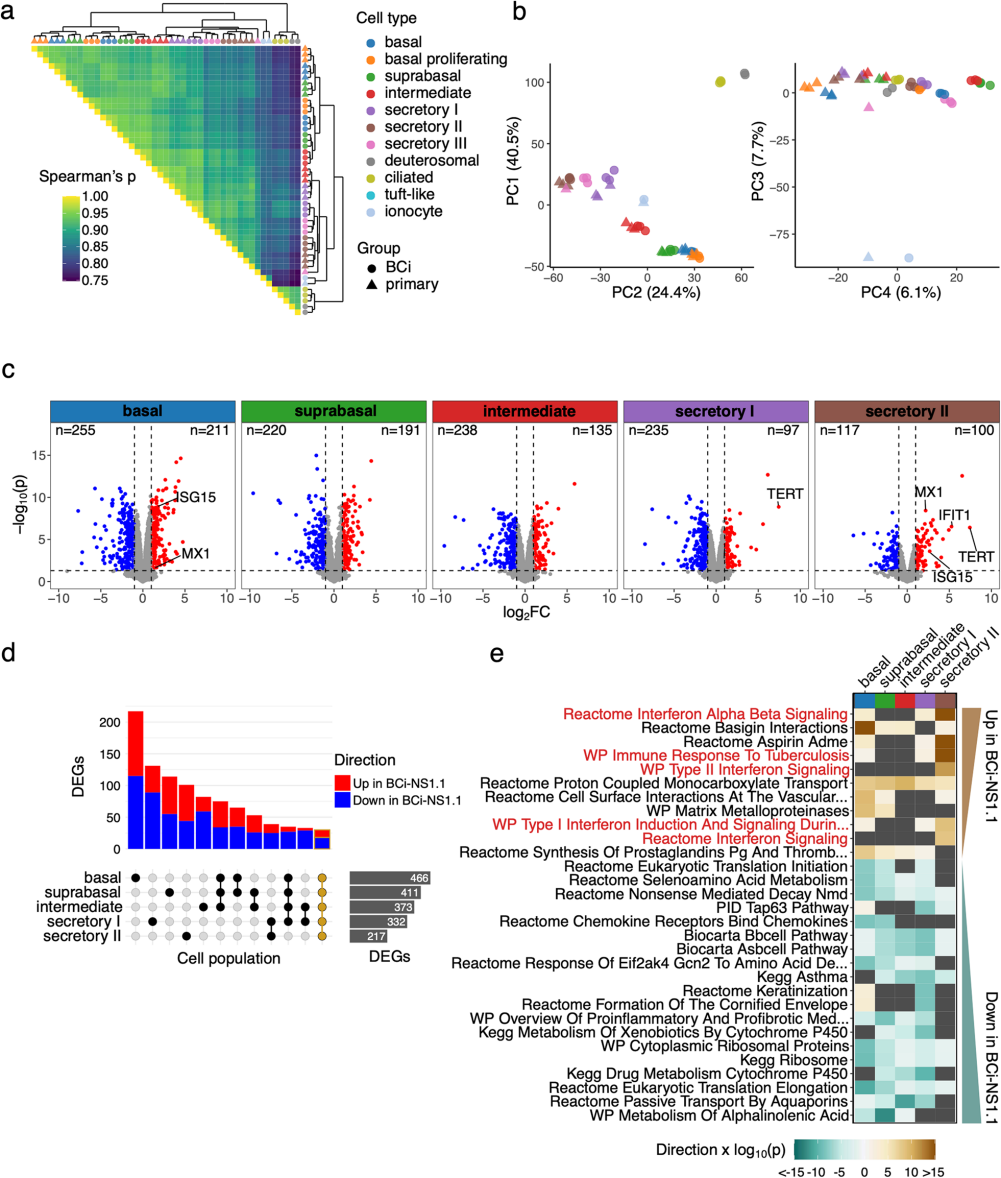
Gene expression analyses of BCi-NS1.1 and primary–derived HAE cultures. **a** Heatmap of Spearman correlation coefficients for pseudobulk transcriptional profiles aggregated by cell population (color) and precursor cell group (shape) by scRNA-Seq replicate. Row and column clustering were determined by Ward’s linkage method and plotted as a marginal dendrogram. **b.** PCA of pseudobulk transcriptional profiles displaying principal components 1 through 4 for the top 3000 most variable genes across plotted samples. The percentage of total variance described by each component is indicated in axis titles. **c**–**e** Differential gene expression analyses contrasting BCi-NS1.1 and primary –derived HAE cultures by cell population for basal, suprabasal, intermediate, secretory I, and secretory II populations. **c** Volcano plots indicating differentially expressed genes (DEGs) in pseudobulk profile contrasts; significance defined as adjusted p-value (Benjamini-Hochberg) < 0.05, log2FC > 1 or < − 1, and per-cell population expression in > 10% of cells. *TERT* and ISGs *IFIT1*, *ISG15*, and *MX1* are labeled when significantly differentially expressed. The number of significant DEGs for each cell population contrast are indicated. **d.** Intersection of DEG lists by cell population. A core set of 30 DEGs across all contrasts is highlighted in gold. **e** The top 30 gene sets enriched in any tested cell population (ranked by FDR, ordered by directional p-value; C2 canonical pathways collection). Dark grey indicates sets that were not significantly enriched. A positive directional p-value indicates enrichment in BCi-NS1.1 relative to primary-derived HAE cultures and a negative value represents enrichment in primary-derived HAE cultures relative to BCi-NS1.1. Gene sets related to interferon signaling are highlighted in red

We next assessed gene expression patterns in each cell population to compare HAE cultures from the primary- and BCi-NS1.1-derived HAE cultures. “Pseudobulk” gene expression profiles were aggregated (by cell population and replicate) and clustered by Spearman correlation distance (Fig. 3a). Data clustered primarily by major cell population (i.e. basal, secretory, ionocyte, ciliated/deuterosomal), and pairwise comparisons further emphasize similarities across the different precursor cell groups by cell population. Interestingly, BCi-NS1.1-derived ‘intermediate’ cell populations clustered with BCi-NS1.1-derived suprabasal cell populations, a result consistent with our pseudotime analysis. Similarly, PCA revealed a strong segregation by cell population in the first three PCs (accounting for 72.6% total variation), with basal/ciliated, basal/secretory, and ionocyte divergence captured by the first three components, respectively (Fig. 3b). PC4 appeared to segregate data by culture type but described a relatively small amount (6.1%) of total variation. Taken together, these results indicate that by gene expression, cell populations are largely similar across BCi-NS1.1 and primary HAE cultures, with a relatively small amount of variation associated with differences in the precursor cell groups.

### Pseudobulk differential gene expression reveals higher interferon-stimulated gene (ISG) expression in some BCi-NS1.1-derived HAE culture cell populations

Despite the high degree of similarity in HAE culture cell populations across precursor cell groups, the relatively minor gene expression differences observed between BCi-NS1.1 and primary-derived HAE culture cell populations in PCA could be biologically meaningful. Therefore, for each cell population, we conducted differential gene expression analysis directly comparing BCi-NS1.1 and primary HAE cultures. In total, we detected 1,052 genes differentially expressed in at least one cell population of those with sufficient cell numbers for testing (basal, suprabasal, intermediate, secretory I, secretory II, Fig. 3c, Additional file 2). A “core” set of 30 genes were detected as differentially expressed (same direction) in all cell populations tested (Fig. 3d). The majority of differentially expressed genes (DEGs) were detected in only one cell population (Fig. 3d). As expected, *TERT* expression was higher in all BCi-NS1.1-derived HAE culture cell populations, though it failed to meet differential expression thresholds in all but secretory I and II cells due to sparse detection typical of droplet scRNA-Seq (Fig. 3c, Fig. S2a). We also compared expression levels of the cell type markers at single cell resolution (Fig. 2b) to the results of pseudobulk contrasts. Notably, although BCi-NS1.1 basal cells exhibit modestly lower expression of *TP63* in the single cell marker gene dot plot (Fig. 2b), this difference (log_2_FC = − 0.53) did not clear differential expression thresholds in pseudobulk contrasts. *MUC5AC* expression was found to be significantly higher in secretory I and II populations from BCi-NS1.1-derived HAE cultures in pseudobulk analyses (consistent with pattern in marker gene dot plot, Fig. 2b), but overall expression was considerably lower than that observed in secretory III cells. Insufficient secretory III cell numbers across biological replicates precluded formal pseudobulk differential expression testing between culture sources.

To place DEGs in biological context, we performed gene set enrichment testing on the C2 canonical pathway gene sets from the Molecular Signatures Database[48]. Several gene sets related to interferon (IFN) signaling were found to be enriched in BCi-NS1.1 cultures, particularly in secretory II cells (Fig. 3e, Additional file 4: Fig. S2a). Relatedly, we observed differential expression of ISGs *MX1*, *IFIT1*, and *ISG15* in secretory II cells and *ISG15* and *MX1* expression in basal cells (indicated in Fig. 3c). Thus, while gene expression is largely similar, a relatively small number of genes are differentially expressed between BCi-NS1.1 and primary-derived HAE culture cell populations. Among these, we observed an elevated ISG signature in some BCi-NS1.1-derived cell populations.

### JAK-STAT pathway activity and expression of select ISGs are higher at steady state in BCi-NS1.1 relative to primary HAE cultures, but are induced to similar levels upon IAV infection

**Fig. 4:**
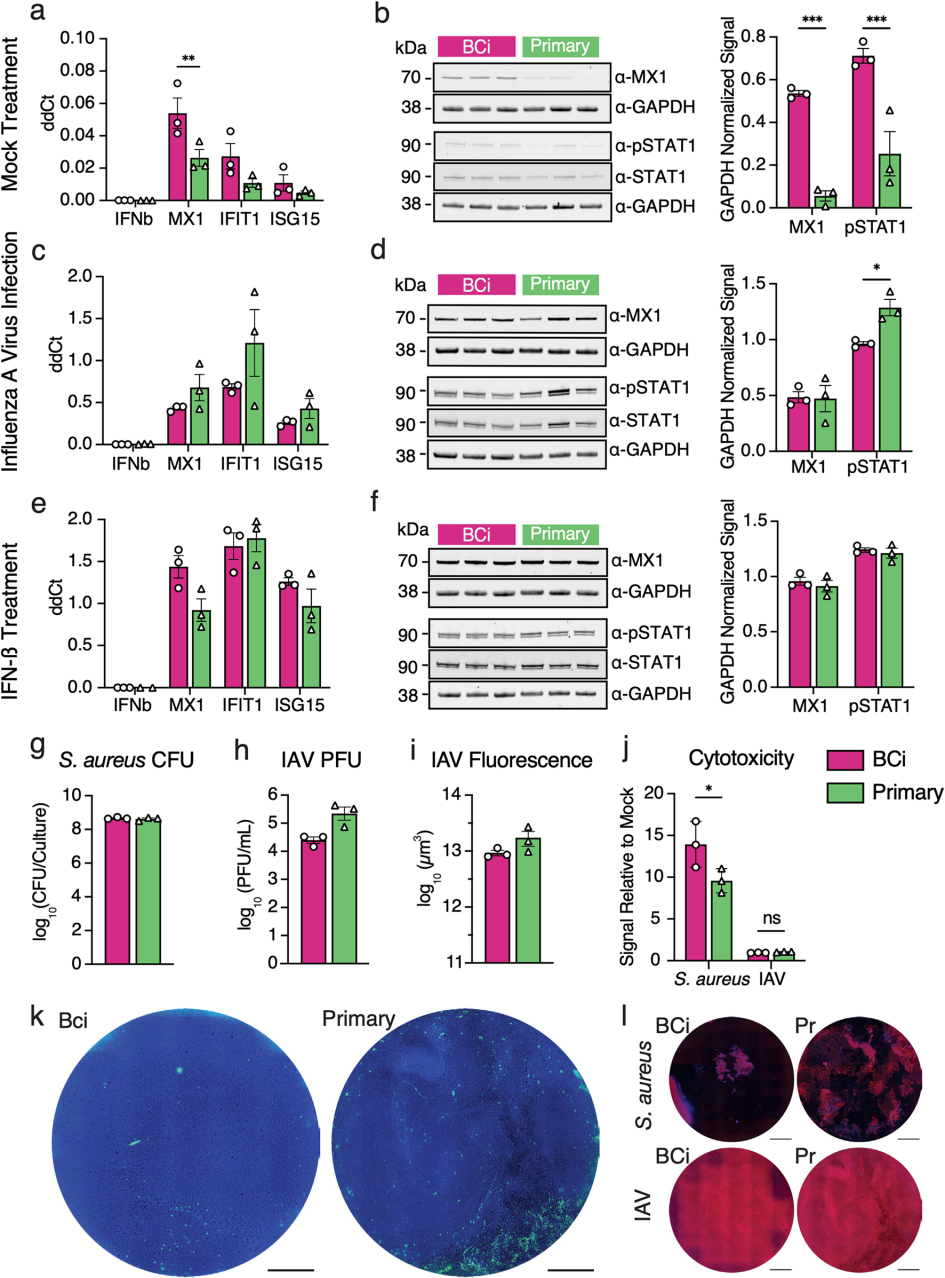
Infection of BCi-NS1.1- and primary-derived HAE cultures with IAV and *S. aureus. ***a**, **c**, **e** RNA levels of MX1, IFN-β, IFIT1 and ISG15 were measured using RT-qPCR at steady-state (**a**), after IAV infection (**c**) and after IFN-β treatment (**e**). **b**, **d**, **f** Representative western blots for MX1 and phosphorylated STAT1 (p-STAT1), along with GAPDH controls for mock treated (**b**), IAV-infected (**d**) and IFN-β-treated (**f**) cultures, and quantification of western blot band intensity from three individual blots for each condition. Blots are cropped to the relevant size for the indicated protein. **g** *S. aureus* colony-forming units (CFU) after 18 h of infection **h** IAV plaque-forming units (PFU) after 24 h of infection **i. **IAV-infected cultures were fixed and labelled with an anti-NP antibody. Infection was quantified as total NP positive area. **j.** LDH release was quantified after IAV or *S. aureus* infection and plotted as a fold change of LDH release over mock infected cultures. **k** Top-down images of representative IAV-infected HAE cultures, showing DAPI (blue) and influenza virus-NP (green). Scale bars = 1 mm. **l.** Top-down images of representative *S. aureus* and IAV-infected HAE cultures, showing Phalloidin (red) and DAPI (blue). Scale bars = 1 mm

As HAE cultures are frequently employed for infection studies by our group and others [8, 15–18, 49] we sought to further characterize potential differences in ISG expression and corresponding signaling between these two culture types at steady state, in response to IFN stimulation, and in response infection with respiratory pathogens. We apically infected both types of cultures with two human respiratory pathogens: IAV and *S. aureus*. As a positive control for ISG induction, we treated cultures basolaterally with human IFN-β. Mock-infected cultures served as controls. We measured the expression of select ISGs (identified in scRNA-Seq analyses) by RT-qPCR and by western blot (Fig. 4a–f). We also measured phosphorylated STAT1 to assess JAK-STAT pathway activity. Mock-treated BCi-NS1.1-derived HAE cultures showed higher expression of ISGs examined (*MX1*, *IFIT1*, *ISG15*) than primary-derived HAE cultures, as observed in our scRNA-Seq analysis (Fig. 3c, e). This difference was statistically significant for *MX1* at both RNA and protein levels (Fig. 4a, b). Concomitantly, phosphorylated STAT1 was elevated in BCi-NS1.1-derived cultures at steady-state (Fig. 4b).

We next sought to determine whether these differences in steady-state ISG expression result in functional differences upon infection with common respiratory pathogens. As expected, we observed mRNA upregulation of ISGs (*MX1*, *IFIT1* and *ISG15*) upon IAV infection (Fig. 4c, d) and IFN-ß treatment (Fig. 4e, f). Interestingly, despite the higher baseline ISG expression in BCi-NS1.1-derived cultures at steady-state, ISG RNA expression was upregulated to similar levels in in primary cell-derived HAE cultures and BCi-NS1.1-derived cultures. MX1 protein levels, while different between culture types at baseline (Fig. 4b), were also induced to similar levels upon IAV infection (Fig. 4d) and IFN-ß treatment (Fig. 4f). Finally, phosphorylated STAT1 levels, while higher in BCi-NS1.1-derived cultures at steady state (Fig. 4b), exhibited an inverse pattern upon IAV infection (Fig. 4c). No difference in phosphorylated STAT1 levels was observed upon IFN-ß stimulation (Fig. 4f). We did observe more variable ISG upregulation (RNA) in primary-derived HAE cultures than in BCi-NS1.1 cell-derived HAE cultures (Fig. 4c, e), possibly due to donor-associated variation.

### Primary and BCi-NS1.1-derived HAE cultures exhibit similar pathogen loads and tissue damage upon infection

We next determined whether the observed increased baseline ISG expression in BCi-NS1.1-derived HAE cultures affects pathogen loads upon infection. Thus, at 24- or 18-h post-infection we measured IAV plaque-forming units (PFU) or *S. aureus* colony-forming units (CFU), respectively, in apical washes from BCi-NS1.1- and primary cell-derived HAE cultures. In addition, we measured pathogen-elicited tissue damage by lactate dehydrogenase (LDH) release and fluorescence microscopy. We found that *S. aureus* loads were equal between both precursor cell types (Fig. 4g). IAV loads trended slightly higher in primary cell-derived HAE cultures (Fig. 4h, l, k, Additional file 4: Fig. S4a), but these differences did not reach statistical significance (p = 0.1 and p = 0.2 for plaque assay and fluorescence data respectively, Mann–Whitney U test). IAV infection resulted in low-level LDH release with no detectable differences across precursor cell types (Fig. 4j). *S. aureus* infection resulted in considerable LDH release, with higher levels in BCi-NS1.1-derived cultures. These patterns were consistent with corresponding fluorescence microscopy of infected cultures to assess cellular damage (Fig. 4l, Additional file 4: Fig. S4b). Taken together, these results demonstrate that, despite some minor differences, BCi-NS1.1- and primary-derived HAE cultures behave similarly upon infection with two major respiratory pathogens, IAV and *S. aureus.*

### BCi-NS1.1-derived HAE cultures express functional CFTR channels

**Fig. 5:**
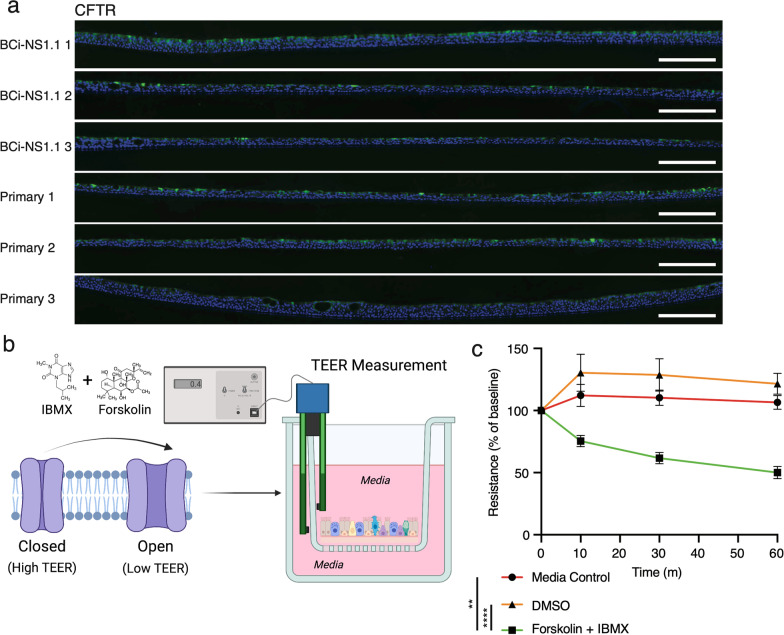
Expression and function of CFTR channels in HAE cultures.** a** Cell type marker immunofluorescence of transections of BCi-NS1.1- or primary-derived HAE cultures. DAPI (nuclei) stained in blue. CFTR labeled in green. Scale bars = 200 µm. **b** Diagrammatic representation of CFTR functional assay. TEER of CFTR channels (purple) was measured at baseline, then activators of CFTR channels or controls were added and TEER was measured at 10–60 min post-treatment. **c** TEER measurement of HAE cultures treated with media control, vehicle control (DMSO) or CFTR channel activator (forskolin and IBMX)

As with any cell culture derived from a single donor, BCi-NS1.1 could exhibit donor-specific properties compatible or incompatible with different research applications. As HAE cultures are widely utilized for studies of airway function in cystic fibrosis, in which dysfunctional CFTR channels result in chronic airway disease, we assessed the expression of CFTR in HAE cultures. In scRNA-Seq data, as expected, mRNA expression of *CFTR* was highest in ionocytes in both BCi-NS1.1-derived and primary derived cultures (Fig. 2b, Additional file 3). Ionocyte *CFTR* mRNA expression levels appeared generally similar across cultures, though insufficient ionocyte numbers precluded a formal statistical comparison. Immunofluorescence labeling (Fig. 5a) confirmed similar CFTR protein expression patterns in both BCi-NS1.1-derived and primary derived cultures. To assess CFTR function in BCi-NS1.1-derived HAE cultures, we measured transepithelial electrical resistance (TEER) of cultures treated with activators of CFTR channels (forskolin and IBMX). Cultures treated with media or vehicle controls exhibited steady TEER over the course of one hour. However, cultures treated with activators of CFTR channels exhibited a decrease in TEER corresponding to CFTR channel opening beginning at 10 min post-activation and extending for up to one hour (endpoint of the experiment). These results indicate that CFTR channels in BCi-NS1.1-derived HAE cultures function similarly to those previously described in primary HAE cultures. [50]

## Discussion

Combining scRNA-Seq, immunofluorescence, and functional analyses, we have shown that HAE cultures derived from the hTERT-expressing airway basal cell line BCi-NS1.1 exhibit similar cell type composition and similar cell type-associated gene expression to HAE cultures derived from primary cells. While we observed evidence of heightened JAK/STAT pathway activity and corresponding increased baseline expression of several ISGs in BCi-NS1.1-derived HAE cultures at steady-state, both precursor cell types induced ISG expression to similar levels upon infection with IAV or upon IFN treatment. IAV infections resulted in apparent but statistically insignificant higher viral loads in primary-derived cultures as compared to BCi-NS1.1-derived cultures. These modest differences are consistent with the elevated steady-state ISG expression observed in BCi-NS1.1-derived cultures, but could also be a consequence of general variation across donor sources. We do not believe that this potential minor dissimilarity should discount the use of BCi-NS1.1 in respiratory virus studies, but should perhaps be considered in infection experiments incorporating multiple culture sources. Finally, we found that BCi-NS1.1-derived HAE cultures express functional CFTR channels, a characteristic important for the study of cystic fibrosis in this model.

Of note, a comprehensive comparison of component cell type gene expression patterns in BCi-NS1.1-derived and primary-derived HAE cultures in this study was somewhat limited due to the unexpectedly low frequency of ciliated cells in our scRNA-Seq data. The underrepresentation of ciliated cells in scRNA-Seq does not reflect the true composition of BCi-NS1.1-derived and primary-derived HAE cultures, but is likely a technical artifact related to cell dissociation and processing prior to loading the 10X Genomics Chromium controller. The proportion of ciliated cells as quantified by villin-1 labeling in our samples is similar across precursor cell types (Fig. 1b, c, e) and is within the expected range for HAE [17, 18]. Indeed, our group and others [17, 18, 22] have effectively detected ciliated cells at expected frequencies by 10X Genomics scRNA-Seq methods. For the unexpectedly few ciliated cells detected in scRNA-Seq data, most of which are from BCi-NS1.1 samples, we observed robust expression of ciliated cell marker genes. In addition, ciliated cells from BCi-NS1.1-derived cultures exhibit highly concordant scRNA-Seq gene expression patterns with ciliated cells from primary-derived HAE cultures in a recently published scRNA-Seq dataset [22]. Although insufficient scRNA-Seq ciliated cell counts precluded a statistically robust differential expression analysis, these observations, along with our additional histological and functional comparisons, are generally consistent with similar ciliated cell features in BCi-NS1.1-derived and primary-derived HAE cultures.

Overall, our results further support BCi-NS1.1-derived HAE cultures as a relevant model system that recapitulates airway epithelial biology similarly to primary-derived HAE cultures. These cultures are of particular benefit in experiments that demand large quantities of HAE cultures/replicates beyond those available from primary cell sources. Additionally, the extended passage capacity of BCi-NS1.1 cells has enabled sophisticated genetic engineering techniques (with corresponding antibiotic selection) for mechanistic studies of airway cell function and disease that can be technically challenging or infeasible in primary cells[26]. Despite overall similarities, we did observe modest differences between culture types. These differences may stem from either donor-to-donor variation, as all BCi-NS1.1-derived cultures were necessarily derived from the same donor while primary-derived cultures were derived from three distinct donors, and/or directly from engineered expression of hTERT in BCi-NS1.1 cells. Future studies in which hTERT expression is engineered in multiple independent primary cell sources may disentangle these mechanisms. Further, determining the specific effects of hTERT in HAE cultures will be important in establishing and interpreting hTERT-engineered culture models of specific demographic groups or disease states for which primary-derived HAE culture systems have been proven highly informative, such as cystic fibrosis[51], COPD[52], and asthma[53].

### Supplementary Information

**Additional file 1:** Cell population frequencies by precursor cell source. Cell frequency data for constituent HAE culture cell populations by culture progenitor group.

**Additional file 2:** Pseudobulk differentially expressed genes across culture progenitor groups. Differentially expressed genes calculated from per-cell type pseudobulk profile contrasts for BCi-NS1.1 vs. primary-derived HAE cultures for.

**Additional file 3:** Marker genes by cell population. Positively expressed marker genes for each cell population calculation from the edgeR GLM.

**Additional file 4: Figure S1.** Additional immunofluorescence labelling of fixed and cross-sectioned HAE cultures from BCi-NS1.1-derived and primary-derived donors. **Figure S2.** scRNA-Seq cell population assignment by replicate and marker gene expression patterns. **Figure S3.** **a** Violin plots of log-normalized expression values for ISGs IFIT1, ISG15, and MX1 along with TERT by cell population. Plots are annotated with the FDR from pseudobulk differential expression testing across BCi-NS1.1-derived and primary HAE cultures where significant. **b** Violin plots of “Hallmark Interferon Alpha Response” gene set scores at single cell resolution by cell population. Low sampling of secretory III, deuterosomal, ciliated cells and ionocytes required their exclusion from pseudobulk contrasts, but they are included here for completeness. **Figure S4.** Top-down images of HAE cultures with individual channels stained for phalloidin (red) and DAPI (blue) and infected with a. IAV (green) and b. S. aureus (green). Scale bars = 1 mm.

## Data Availability

scRNA-Seq data generated for this study are available via the NCBI Gene Expression Omnibus repository with accession number GSE225765. All analysis code is available on GitHub at https://github.com/BradRosenbergLab/airwayepithelialculturecomparison.
