# Supplementary material for: A comparative study of in vitro air–liquid interface culture models of the human airway epithelium evaluating cellular heterogeneity and gene expression at single cell resolution
Source: Respir Res. 2023 Aug 28;24:213. doi: 10.1186/s12931-023-02514-2 (PMC10464153; doi:10.1186/s12931-023-02514-2)
Supplement: Supplementary file 4 — Additional file 4: Figure S1. Additional immunofluorescence labelling of fixed and cross-sectioned HAE cultures from BCi-NS1.1-derived and primary-derived donors. Figure S2. scRNA-Seq cell population assignment by replicate and marker gene expression patterns. Figure S3. a Violin plots of log-normalized expression values for ISGs IFIT1, ISG15, and MX1 along with TERT by cell population. Plots are annotated with the FDR from pseudobulk differential expression testing across BCi-NS1.1-derived and primary HAE cultures where significant. b Violin plots of “Hallmark Interferon Alpha Response” gene set scores at single cell resolution by cell population. Low sampling of secretory III, deuterosomal, ciliated cells and ionocytes required their exclusion from pseudobulk contrasts, but they are included here for completeness. Figure S4. Top-down images of HAE cultures with individual channels stained for phalloidin (red) and DAPI (blue) and infected with a. IAV (green) and b. S. aureus (green). Scale bars = 1 mm. [file 12931_2023_2514_MOESM4_ESM.pdf]

Supplementary Material:

A comparative study of in vitro air-liquid interface culture models of the human airway epithelium evaluating cellular heterogeneity and gene expression at single cell resolution

Rachel A. Prescott[2*], Alec P. Pankow[1*], Maren de Vries[2*], Keaton Crosse[2], Roosheel S. Patel[1], Mark Alu[3], Cindy Loomis[3], Victor Torres[2], Sergei Koralov[3], Ellie Ivanova[3], Meike Dittmann[2+], Brad R. Rosenberg[1+]

Affiliation

[1] Department of Microbiology, The Icahn School of Medicine at Mount Sinai
[2] Department of Microbiology, NYU Grossman School of Medicine
[3] Department of Pathology, NYU Grossman School of Medicine

[*]Equal contribution
[+]Co-corresponding author

Corresponding Author(s):

Meike Dittmann, Ph.D.
meike.dittmann@nyulangone.org

Brad R. Rosenberg, M.D., Ph.D.
brad.rosenberg@mssm.edu

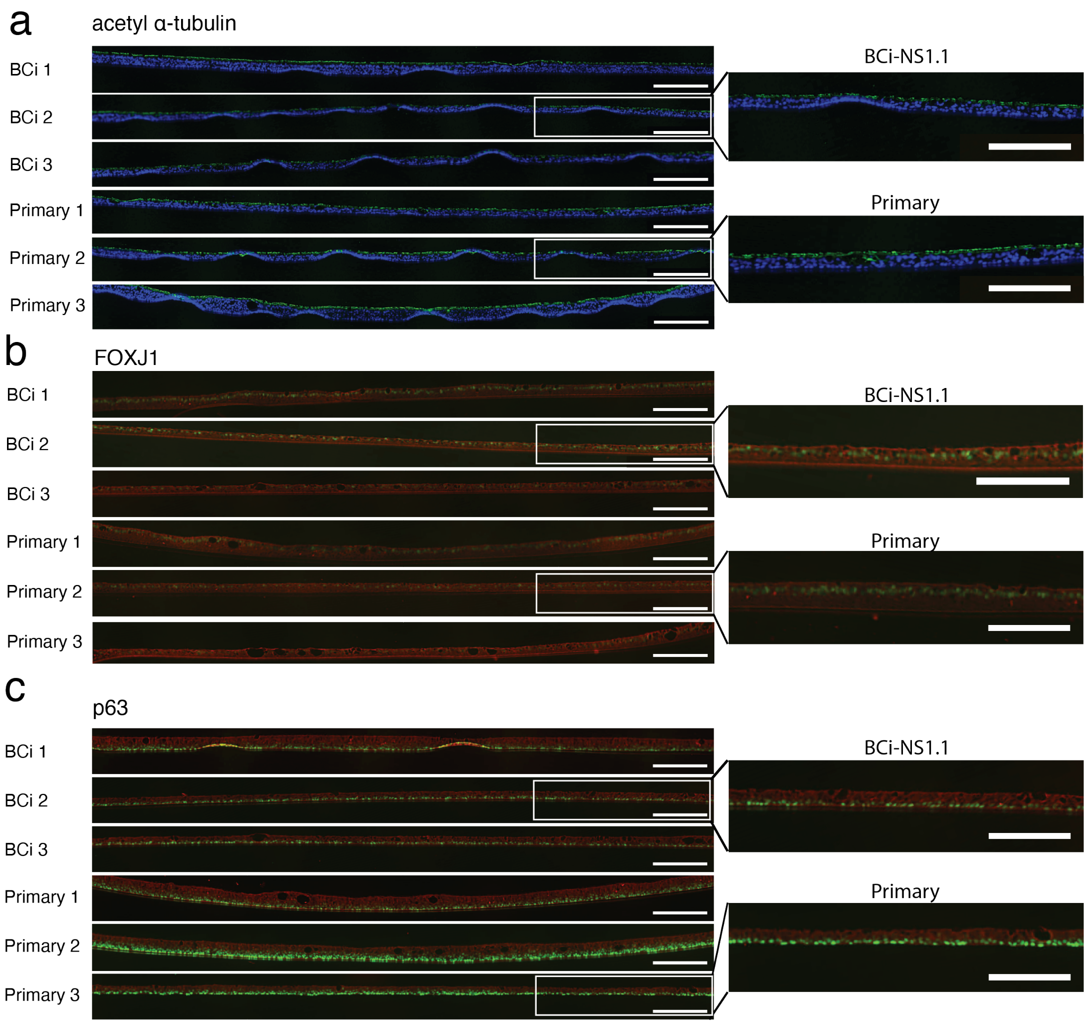

Supplementary Figure 1: Additional immunofluorescence labelling of fixed and cross-sectioned HAE cultures from BCi-NS1.1-derived and primary-derived donors. Scale bars = 200µm (left, full transections from each donor) and 150µm (right, representative zoomed in images from each progenitor cell type). Phalloidin (red), DAPI (blue) a. aa-tubulin (green) b. FOXJ1 (green) and c. TP63 (green)

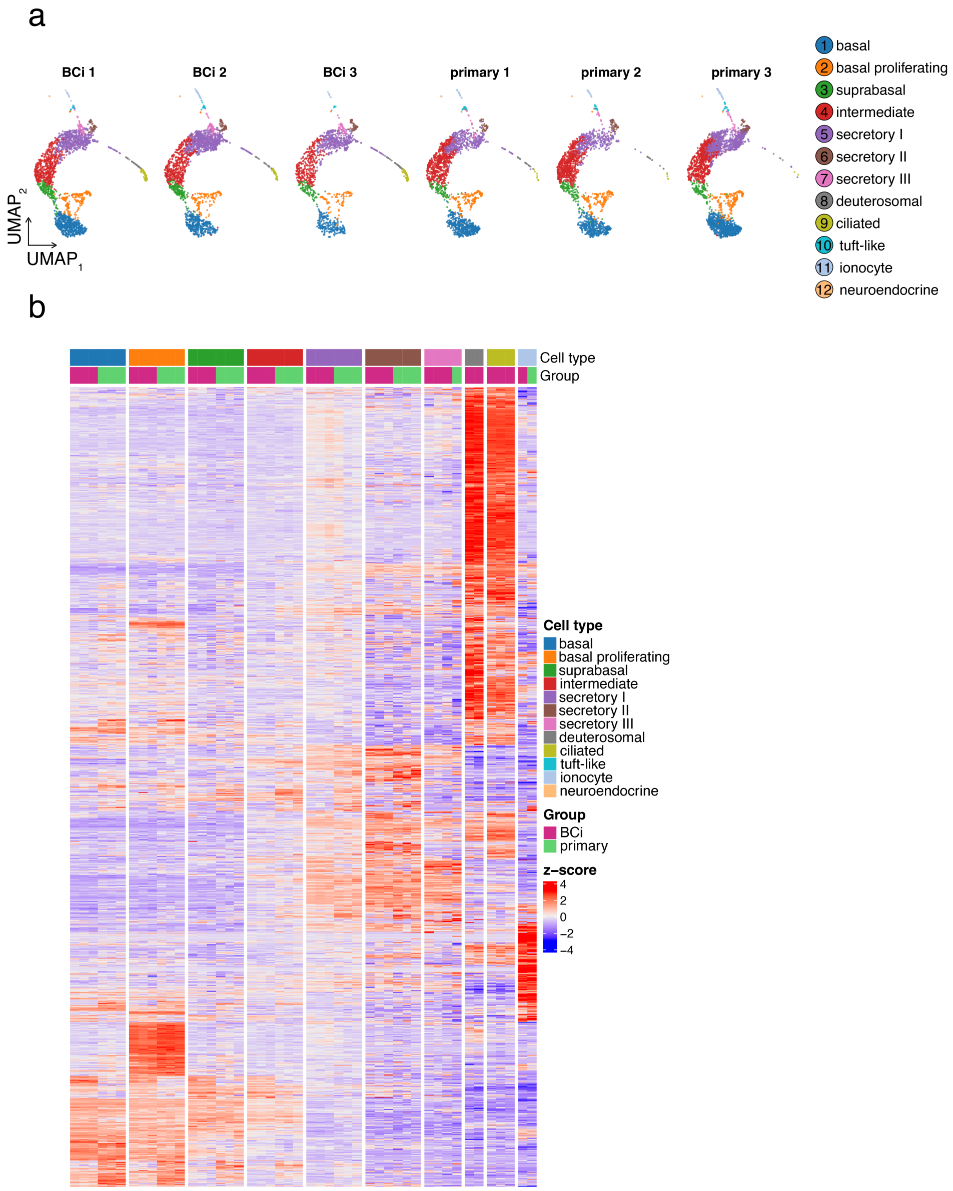

Supplementary Figure 2: scRNA-Seq cell population assignment by replicate and marker gene expression patterns. a. UMAP dimensionality reduction plots of scRNA-Seq independently normalized with scTransform (v.0.3.3) and integrated using Seurat 4.0 commands *PrepSCTIntegration*, *FindIntegrationAnchors* and *IntegrateData*, displaying uniformity in cell type annotation across replicates. **b.** Heatmap of 4,001 marker genes by cell population from scRNA-seq, with rows clustered by enrichment pattern and column ordering by cell and progenitor type. For a list of all marker genes, see Supplementary Data File 2. Differentially expressed markers were identified by contrasting profiles within a cell population with the average expression of remaining cell annotations, using significance cutoffs of $Log_2FC > 2$ (positively expressed only) and an FDR of < 0.05.

a

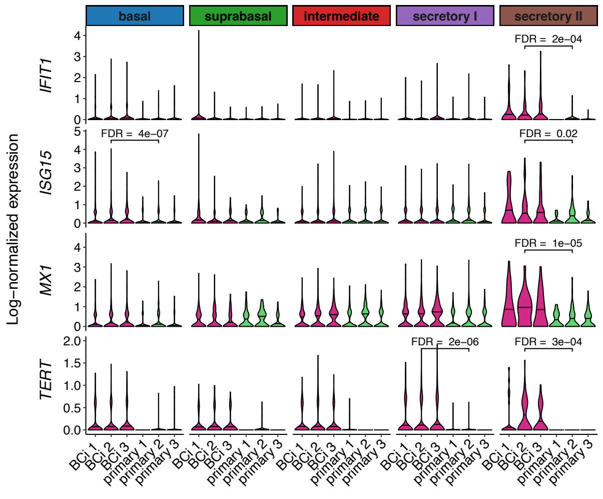

b

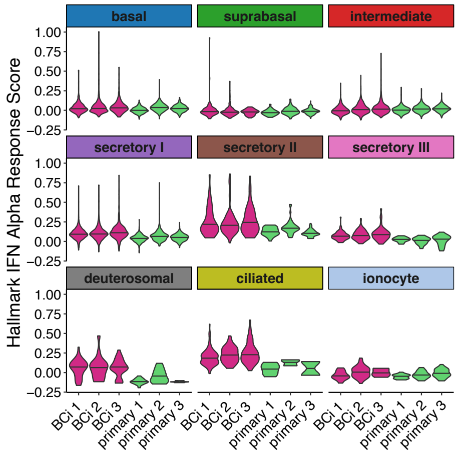

Supplementary Figure 3: a. Violin plots of log-normalized expression values for ISGs *IFIT1*, *ISG15*, and *MX1* along with *TERT* by cell population. Plots are annotated with the FDR from pseudobulk differential expression testing across BCi-NS1.1-derived and primary HAE cultures where significant. **b.** Violin plots of “Hallmark Interferon Alpha Response” gene set scores at single cell resolution by cell population. Low sampling of secretory III, deuterosomal, ciliated cells and ionocytes required their exclusion from pseudobulk contrasts, but they are included here for completeness.

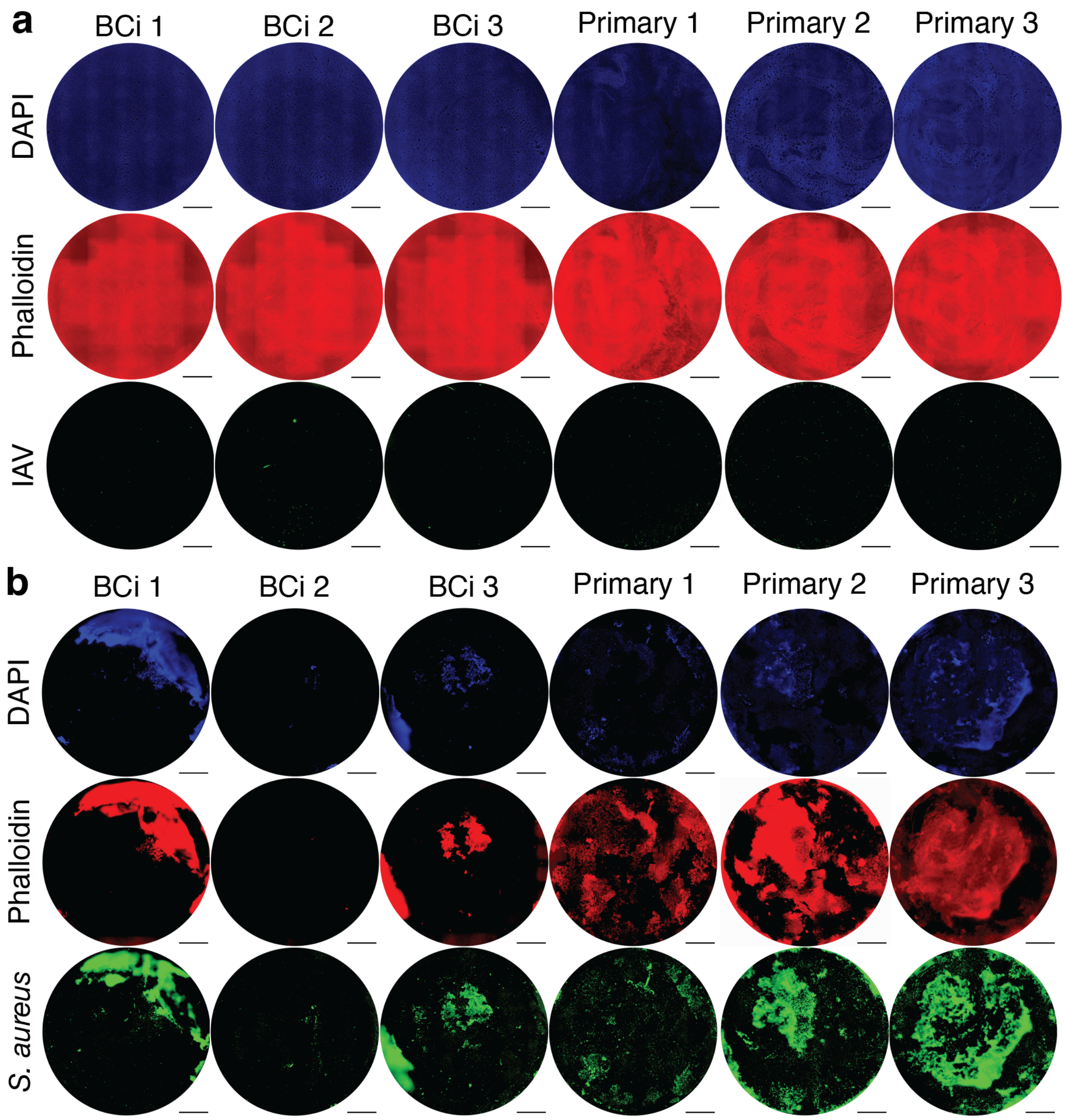

Supplementary Figure 4: Top-down images of HAE cultures with individual channels stained for phalloidin (red) and DAPI (blue) and infected with **a.** IAV (green) and **b.** *S. aureus* (green). Scale bars = 1mm.
